# Lung cancer stage at diagnosis and immigrant English/French language proficiency: a retrospective population level cohort study of urban residents in Ontario, Canada

**DOI:** 10.1186/s12885-025-14666-z

**Published:** 2025-09-30

**Authors:** Jennifer Zhong, Xiaoxuan Han, Aisha Lofters, Jastaranpreet Singh, Geetanjali Datta

**Affiliations:** 1 Department of Human Biology, University of Toronto, Toronto, Canada; 2 WHO Collaborating Centre for Infectious Disease Epidemiology and Control, School of Public Health, Li Ka Shing Faculty of Medicine, The University of Hong Kong, Pokfulam, Hong Kong, Hong Kong S.A.R., China; 3 Dalla Lana School of Public Health, Biostatistics Division, University of Toronto, Toronto, Canada; 4 Department of Family & Community Medicine, University of Toronto, Toronto, Canada; 5 Peter Gilgan Centre for Women’s Cancers, Women’s College Hospital, Toronto, Canada; 6 Department of Immunology, University of Toronto, Toronto, Canada; 7 Cedars-Sinai Cancer Research Center for Health Equity, Los Angeles, USA; 8 Department of Biomedical Sciences, Cedars-Sinai Medical Center, Los Angeles, USA

**Keywords:** Lung cancer, Screening, Immigrant health, Language fluency

## Abstract

**Background:**

Lung and bronchial cancer is the most diagnosed cancer among both sexes in Canada and has one of the lowest survival rates.

**Methods:**

This population level retrospective cohort study examined the associations between the lung cancer stage at diagnosis and English/French fluency. The study used multiple linked health-administrative databases to create a cohort of urban-dwelling Ontarian immigrants and long-term residents aged 45–105 diagnosed with incident lung cancer between 1 January 2010 and 31 December 2020. Modified Poisson regression was used to examine the risk of diagnosis at early vs. late stages among immigrants who do not speak English or French fluently compared with immigrants who speak English or French fluently. The fully adjusted model included age, sex, neighborhood-area income quintile, lung cancer type, number of primary care visits prior to diagnosis, and region of origin.

**Results:**

Approximately 57.7% of the 96,613 people diagnosed with incident lung cancer between 2010 and 2020 were diagnosed at the late stage. Non-English/French fluent immigrants were no more likely to be diagnosed at a late stage than English/French fluent immigrants and long-term residents (57.6% vs. 57.8% and 57.7%). However, in fully adjusted models, people living in lower neighborhood income quintiles were more likely to be diagnosed at a late stage (e.g., income quintile 1 [lowest] vs. quintile 5 [highest]: [ARR 1.08; 95% CI: 1.02–1.15]), as were immigrants from the Caribbean [ARR 1.16; 95% CI: 1.05–1.29] and South Asia [ARR 1.10; 95% CI: 1.02–1.19].

**Conclusions:**

Although lung cancer is frequently diagnosed at a late stage in Ontario and we found socioeconomic inequalities, fluency in Canada’s official languages was not associated with late diagnosis in this study.

**Supplementary Information:**

The online version contains supplementary material available at 10.1186/s12885-025-14666-z.

## Background

Lung and bronchial cancer is the most diagnosed cancer among both sexes in Canada, with approximately 13% of new cases diagnosed in 2021 [1]. Unfortunately, over 50% of new lung cancer cases are diagnosed at stage IV, with a 5% survival rate [2]. As survival rates can vary from over 70% at stage I to 5% at stage IV [2], early detection is key to reducing lung and bronchus cancer death. A key barrier to early detection is that lung cancer symptoms are often difficult to detect or easily mistaken for other conditions. Lofters et al.. (2021) noted that lung cancer is often diagnosed symptomatically in Canada, as screening programs are still not yet widespread, leading to delays in diagnosis [3]. This highlights the need for the creation of equitable lung cancer screening programs.

Research has shown that inequalities in cancer diagnosis exist [4–12]. Some factors affecting the timing of diagnosis are socioeconomic status, healthcare, and immigration related variables. System delays, particularly referrals to treatment and wait times for chemotherapy, radiotherapy or surgery, have been shown to have a major effect on cancer diagnosis and long-term prognosis [13–15]. Immigrants have been identified as a vulnerable population, as they are disproportionately affected by poverty, food and housing insecurity, stigma and marginalization, access to primary care and more [16]. Work by co-authors on this paper in Ontario, Canada showed no differences in stage of lung cancer diagnosis for immigrants [3]. However, an additional factor that has rarely been studied in the context of cancer diagnosis is official language fluency. Canada has two official languages: English and French. However, in the 2021 census, approximately one in four Canadians had a mother tongue other than English or French, and nearly one in eight predominantly spoke a language other than English or French at home [17]. Although official language fluency has been studied with respect to healthcare access [18–21], there are gaps in the literature relating language fluency to disparities in cancer diagnosis.

In this population level retrospective cohort study, building on previous work [3], we used population-based health and administrative data in Ontario, Canada to explore stage of lung cancer diagnosis (dichotomized as early vs. late) between urban Ontario immigrants who did not speak English or French fluently at the time of landing and urban Ontario immigrants who did speak English or French fluently, and to examine the association of the stage of diagnosis with potentially actionable sociodemographic and health-related factors.

## Methods

### Data sources

Methods described are similar to our previous work [3]. We used several databases available at ICES (formerly known as the Institute for Clinical Evaluative Sciences), an independent, non-profit research institute holding the most comprehensive set of linked, health-related data in Ontario. As a prescribed entity under Ontario’s privacy legislation, ICES is authorized to collect and use healthcare data for the purposes of health system analysis, evaluation, and decision support. Secure access to this data is governed by policies and procedures that are approved by the Information and Privacy Commissioner of Ontario. All datasets were linked via unique encrypted identifiers and analyzed at ICES.

The Immigration, Refugee and Citizenship Canada Permanent Resident (IRCC-PR) database contains detailed demographic information on immigrants and refugees who landed in Ontario from 1985 onward, including country of birth, date of landing, and English/French language proficiency. The Ontario Cancer Registry (OCR) documents data on all Ontario residents who have an incident cancer diagnosed in Ontario or who have died from cancer (with the exception of non-melanoma skin cancers) since 1964, including the diagnosis date, age at diagnosis, stage at diagnosis, histology, and cause of death if applicable. The TNM Classification of Malignant Tumors (TNM) staging system, a globally recognized standard for classifying the spread of malignant tumors, was used to determine the stage at diagnosis. If the TNM stage was unavailable, physician staging from the regional cancer center was used. If neither was captured (due to limited stage work-up and/or limited documentation in the patient’s medical record), the stage was coded as missing. The Ontario Health Insurance Plan (OHIP) database contains all OHIP insured medical services received by a patient, including the date, healthcare provider, and location.

### Study cohort

Since 90% of immigrants to Ontario live in urban settings [22], we included all urban-dwelling Ontarians aged 45–105 years of age with a new diagnosis of lung cancer as determined from OCR records between 1 January 2010 and 31 December 2020 with a unique ICES identifier. The study included lung cancer diagnosed in those younger that the typical lung cancer screening age of 55 years because lung cancer screening is in its pilot stages in Ontario [23] and there has been a recent up-tick in cancer diagnoses in those younger than 50 years of age in Canada [24]. We excluded anyone whose cancer was either stage 0 or in situ at the time of diagnosis, who had no contact with the healthcare system for three years prior to their date of diagnosis, and who became eligible for provincial health insurance after their diagnosis date. We also excluded those who were diagnosed with lung cancer at less than 45 years of age, as this group may have a distinct disease process. We excluded those in the IRCC-PR database without self-reported English/French language proficiency data upon landing (0.003% of all immigrants). Finally, we categorized the cohort into long-term residents (those not included in the IRCC- PR database and thus either Canadian-born or immigrants who arrived prior to 1985) and immigrants (those included in the IRCC-PR database and thus arrived after 1985).

### Variables

For each patient, we determined sex, age, and neighborhood income quintile at the time of diagnosis from relevant databases. Cancer types were categorized as small cell, squamous cell, adenocarcinoma, and other. To describe primary care contact, we split the number of visits to a primary care physician for each patient in the 0–24 months before diagnosis (a two-year period) into quartiles, with level one being the lowest and level four being the highest number of visits. We further subcategorized immigrants by region of origin on the basis of their country of birth.

### Exposure

“Non-English/French speaking immigrants” and “English/French speaking immigrants” on the basis of self-reported language data upon landing from the IRCC-PR.

### Outcome

The outcomes were dichotomous: early (I–II) vs. late (III–IV) stage diagnosis based on OCR staging.

### Analysis

We described the cohort by immigration and official language proficiency and by the variables above. We further described each immigrant language proficiency group by early vs. late stages of diagnosis and the variables above. Standardized differences were calculated to compare variable distributions between groups. For each variable, the standardized difference was determined by taking the difference in proportions between groups and dividing it by the pooled standard deviation of these proportions. A standardized difference greater than 0.10 was considered clinically significant in these comparisons.

For our multivariate Poisson regression model, we limited the cohort to immigrants and refugees with non-missing cancer staging. We employed Poisson regression models with robust standard errors to explore the risks of early vs. late stage lung cancer diagnosis among the immigrant cohorts; first unadjusted, then adjusted for age and sex (with age as a categorical variable), then fully adjusted for age, sex, neighborhood income quintile, cancer type, number of prior primary care visits and region of origin. Relative risks were calculated within each model to measure the association between language proficiency and the stage of lung cancer diagnosis while controlling for confounding variables. We conducted statistical analysis via SAS software (9.4). The study was approved by the Research Ethics Board at the University of Toronto.

As language ability was assessed upon arrival in Canada, we investigated whether the length of time since immigration influenced the stage at which lung cancer was diagnosed. We categorized the time since arrival into four different levels using quartiles, and logistic regression was used to estimate the odds of late-stage diagnosis across quartile levels. The model parameters were estimated using maximum likelihood estimation (MLE). We calculated the odds ratios to compare the likelihood of late-stage diagnosis among newly arrived immigrants (level 0) against those who had been in the country the longest (level 4).

## Results

**Fig. 1:**
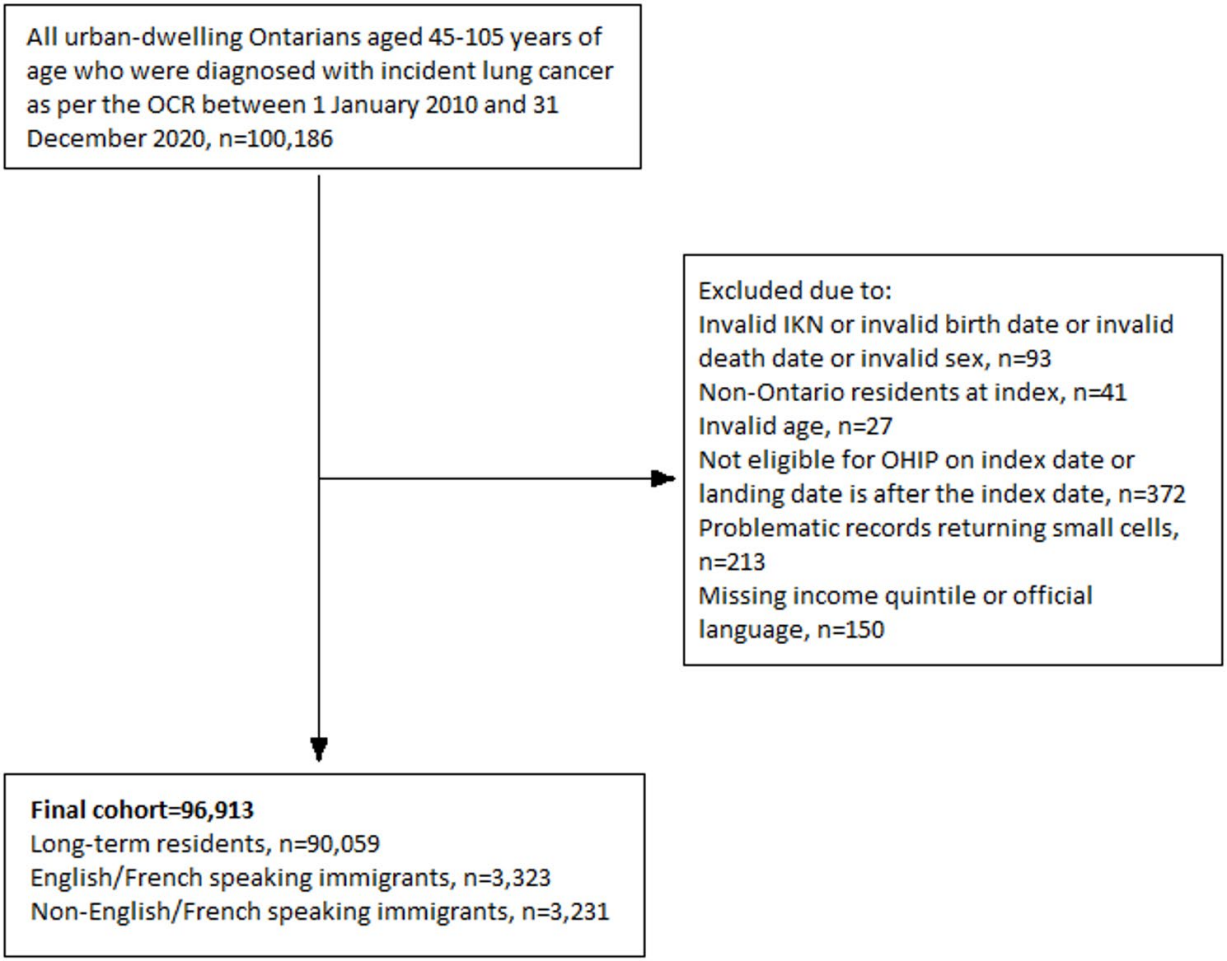
Cohort creation

Between January 1, 2010, and December 31, 2020, a total of 100, 186 people were diagnosed with incident lung cancer in Ontario, Canada (Fig. 1). 96,913 people were included in this study, 6,554 (6.8%) of whom were immigrants were diagnosed with incident lung cancer in Ontario, Canada (Table 1). The English/French speaking group was diagnosed at younger ages than the non-English/French speaking group (48.0% diagnosed at < 65 years versus 30.8%) and were less likely to be female (39.2% vs. 43.0%). Non-English/French speaking immigrants were more likely to be diagnosed with squamous cell carcinoma (12.6% vs. 9.7%) and less likely to be diagnosed with adenocarcinoma (38.8% vs. 42.4%) than English/French speaking immigrants. Both immigrant groups were more likely to belong to the lowest neighborhood income quintiles and had more primary care visits than long-term residents did. No notable differences were observed in the region of origin (Table 2).

**Table 1:** Descriptive characteristics by immigrant status of 96,613 people residing in Ontario who were diagnosed with an incident lung cancer as per the OCR at any time between 1 January 2010 and 31 December 2020

Characteristics	Long-term Residents (*n *= 90,059)	English/French Speaking Immigrants (*n *= 3,323)	Non-English/French Speaking Immigrants (*n *= 3,231)	S.D. (Non-English/French speaking immigrants – English/French speaking immigrants)	Total (*n *= 96,613)
**Sex**					
Female	44,915 (49.9%)	1,301 (39.2%)	1,388 (43.0%)	0.08	47,604 (49.3%)
Male	45,144 (50.1%)	2,022 (60.9%)	1,843 (57.0%)	0.08	49,009 (50.7%)
**Age Group at Diagnosis**					
45-54	4,971 (5.5%)	550 (16.6%)	304 (9.3%)	0.22	5,822 (6.0%)
55-64	18,504 (20.6%)	1,043 (31.4%)	695 (21.5%)	0.23	20,242 (21.0%)
65-74	30,470 (33.8%)	934 (28.1%)	929 (28.8%)	0.01	32,333 (33.5%)
75-84	26,788 (29.7%)	610 (18.4%)	985 (30.5%)	0.29	28,383 (29.4%)
85+	9,326 (10.4%)	186 (5.6%)	321 (9.4%)	0.16	9,833 (10.2%)
**Neighborhood Income Quintile**					
Quintile 1 (lowest)	21,743 (24.1%)	957 (28.8%)	976 (30.2%)	0.03	23,767 (24.5%)
Quintile 2	20,358 (22.6%)	763 (23.0%)	736 (22.8%)	0.00	21,857 (22.6%)
Quintile 3	17,502 (19.4%)	636 (19.1%)	573 (17.7%)	0.04	18,711 (19.4%)
Quintile 4	15,793 (17.5%)	544 (16.4%)	564 (17.5%)	0.03	16,901 (17.5%)
Quintile 5 (highest)	14,663 (16.3%)	423 (12.7%)	382 (11.8%)	0.03	15,468 (16.0%)
**Lung Cancer Type**					
Adenocarcinoma	27,849 (30.9%)	1,409 (42.4%)	1,255 (38.8%)	0.07	30,513 (31.6%)
Others	39,470 (43.8%)	1,362 (41.0%)	1,321 (40.9%)	0.00	42,153 (43.6%)
Small Cell	9,292 (10.3%)	231 (7.0%)	247 (7.6%)	0.03	9,770 (10.1%)
Squamous	13,448 (14.9%)	321 (9.7%)	408 (12.6%)	0.09	14,177 (14.7%)
**Region of Origin**					
Caribbean	-	250 (7.5%)	8 (0.25%)	0.38	258 (3.9%)
Central America	-	22 (0.7%)	23 (0.71%)	0.01	45 (0.7%)
East Africa	-	94 (2.8%)	15 (0.5%)	0.19	109 (1.7%)
East Asia	-	554 (16.7%)	1,321 (40.9%)	0.56	1,875 (28.6%)
Eastern Europe	-	195 (5.9%)	447 (13.8%)	0.27	642 (9.8%)
Middle East	-	223 (7.0%)	198 (6.1%)	0.04	431 (6.6%)
North Africa	-	35-39	1<6	0.15	39 (0.6%)
North America	-	127 (3.8%)	0 (0.0%)	0.28	127 (1.9%)
South America	-	164 (4.9%)	51 (1.6%)	0.19	215 (3.3%)
South Asia	-	437 (13.2%)	337 (10.4%)	0.08	774 (11.8%)
Southeast Asia	-	609 (18.3%)	326 (10.1%)	0.24	935 (14.3%)
Southern Africa	-	14 (0.4%)	0 (0.0%)	0.09	14 (0.2%)
Southern Europe	-	75 (2.3%)	115 (3.6%)	0.08	190 (2.9%)
USSR (former)	-	165 (5.0%)	186 (5.8%)	0.04	351 (5.4%)
United Kingdom	-	<6	0	0.03	<6
Western Africa	-	5-10	0 (0.0%)	0.06	5-10
Western Europe	-	227 (6.8%)	15 (0.5%)	0.34	242 (3.7%)
Yugoslavia (former)	-	110 (3.3%)	188 (5.8%)	0.12	298 (4.6%)
**No. PCP visits 0–24 months < index—patient’s usual provider of care**					
Mean +- SD	24.1+−24.0	27.4+−25.1	27.4+−23.8	0.00	24.3+−24.0

**Institutional data requirements do not allow the reporting of any cell size less than 6, or reporting of any cell that would allow the calculation of another cell with size of less than 6

**Table 2:** Descriptive characteristics by Language status of 6,554 immigrants residing in Ontario who were diagnosed with an incident lung cancer as per the OCR at any time between 1 January 2010 and 31 December 2020

Characteristics	English/French Speaking (*n *= 3,323)	Non-English/French Speaking (*n *= 3,231)	Standardized Difference (Non-English/French speaking – English/French speaking)	Total (*n *= 6,554)
**Age Group at Diagnosis^**				
45-54	550 (16.6%)	304 (9.3%)	0.22	854 (13%)
55-64	1,043 (31.4%)	695 (21.5%)	0.23	1,738 (26.5%)
65-74	934 (28.1%)	929 (28.8%)	0.01	1,863 (28.4%)
75-84	610 (18.4%)	985 (30.5%)	0.29	1,595 (24.3%)
85+	186 (5.6%)	321 (9.4%)	0.16	507 (7.7%)
**Neighborhood Income Quintile^**				
Quintile 1 (lowest)	957 (28.8%)	976 (30.2%)	0.03	1,933 (29.8%)
Quintile 2	763 (23.0%)	736 (22.8%)	0.00	1,499 (22.9%)
Quintile 3	636 (19.1%)	573 (17.7%)	0.04	1,209 (18.4%)
Quintile 4	544 (16.4%)	564 (17.5%)	0.03	1,108 (16.9%)
Quintile 5 (highest)	423 (12.7%)	382 (11.8%)	0.03	805 (12.2%)
**Region of Origin^**				
Caribbean	250 (7.5%)	8 (0.25%)	0.38	258 (3.9%)
Central America	22 (0.7%)	23 (0.71%)	0.01	45 (0.7%)
East Africa	94 (2.8%)	15 (0.5%)	0.19	109 (1.7%)
East Asia	554 (16.7%)	1,321 (40.9%)	0.56	1,875 (28.6%)
Eastern Europe	195 (5.9%)	447 (13.8%)	0.27	642 (9.8%)
Middle East	223 (7.0%)	198 (6.1%)	0.04	431 (6.6%)
North Africa	35-39	1<6	0.15	39 (0.6%)
North America	127 (3.8%)	0 (0.0%)	0.28	127 (1.9%)
South America	164 (4.9%)	51 (1.6%)	0.19	215 (3.3%)
South Asia	437 (13.2%)	337 (10.4%)	0.08	774 (11.8%)
Southeast Asia	609 (18.3%)	326 (10.1%)	0.24	935 (14.3%)
Southern Africa	14 (0.4%)	0 (0.0%)	0.09	14 (0.2%)
Southern Europe	75 (2.3%)	115 (3.6%)	0.08	190 (2.9%)
USSR (former)	165 (5.0%)	186 (5.8%)	0.04	351 (5.4%)
United Kingdom	<6	0 (0.0%)	0.03	<6
Western Africa	5-10	0 (0.0%)	0.06	5-10
Western Europe	227 (6.8%)	15 (0.5%)	0.34	242 (3.7%)
Yugoslavia (former)	110 (3.3%)	188 (5.8%)	0.12	298 (4.6%)
**No. PCP visits 0-24 months < index—patient’s usual provider of care^**				
Mean ± SD	27.4 ± 25.1	27.4 ± 23.8	0.00	27.4 ± 24.5
**Years since Landing**				
Mean ± SD	18.0 ± 8.2	17.3 ± 8.3	-	17.7 ± 8.3
Median (IQR)	19.1 (12.0, 24.3)	18.2 (11.2, 23.7)	-	18.6 (11.7, 23.9)
Level 0 (0 to ≤ 9.7 years)	710 (10.8%)	601 (9.2%)	0.05	1311 (20%)
Level 1 (> 9.7 to ≤ 16.2 years)	667 (10.2%)	642 (9.8%)	0.01	1309 (20%)
Level 2 (> 16.2 ≤ 20.7 years)	661 (10.1%)	651 (9.9%)	0.007	1312 (20%)
Level 3 (> 20.7 to ≤ 25.2 years)	669 (10.2%)	642 (9.8%)	0.01	1311 (20%)
Level 4 (> 25.2 to ≤ 35.4 years)	616 (9.4%)	895 (10.6%)	0.04	1311 (20%)

**Institutional data requirements do not allow the reporting of any cell size less than 6, or reporting of any cell that would allow the calculation of another cell with size of less than 6

^Variables included in the full adjusted model

In the overall cohort, 24.5% of the patients were diagnosed at an early stage, 57.7% at a late stage, and 17.8% were missing staging data. The stage of diagnosis did not differ significantly between the two immigrant groups with Non-English/French speaking immigrants being diagnosed at 22.5%, 57.6%, and 19.7% respectively versus English/French speaking immigrants at 23.0%, 57.8%, and 19.1%, respectively (Table 3). For both groups, men were more likely to be diagnosed at a later stage, with only 45.5% of men being diagnosed at an early stage. For both groups, late-stage diagnosis was associated with a lower neighborhood income quintile, younger age group, and lower number of primary care visits (< 10). In both groups, patients diagnosed at a late stage were more likely to have small cell carcinoma. The stage of diagnosis was not associated with the region of origin.

**Table 3:** Descriptive characteristics of immigrant cohort (excluding patients with missing lung cancer stage) stratified by stage of diagnosis (early vs. late) and Language proficiency

Characteristics	Standardized Difference (Non English/French speaking – English/French speaking	English/French Speaking Immigrants (*n *= 2,687)		Standardized Difference (Late – Early stage for English/French speaking)	Non-English/French Speaking Immigrants (*n *= 2,594)		Standardized Difference Late – Early stage for Non-English/French speaking)
		Early Stage (*n *= 774)	Late Stage (*n *= 1,913)		Early Stage (*n *= 740)	Late Stage (*n *= 1,854)	
**Age Group^**							
45-54	0.09	120 (15.5%)	355 (18.6%)	0.13	72 (9.7%)	187 (10.1%)	0.13
55-64	0.12	230 (29.7%)	625 (32.7%)	0.07	153 (20.7%)	426 (23.0%)	0.07
65-74	0.01	239 (30.9%)	507 (26.5%)	0.08	230 (31.1%)	514 (27.7%)	0.08
75-84	0.08	155 (20.0%)	332 (17.4%)	0.06	228 (30.8%)	569 (30.7%)	0.06
85+	0.11	30 (3.9%)	94 (4.9%)	0.07	57 (7.7%)	158 (8.5%)	0.07
**Neighborhood Income Quintile^**							
Quintile 1 (lowest)	0.02	228 (29.5%)	555 (29.0%)	0.01	222 (30.0%)	564 (30.4%)	0.01
Quintile 2	0.02	162 (20.9%)	466 (24.4%)	0.08	165 (22.3%)	450 (24.2%)	0.08
Quintile 3	0.01	137 (17.7%)	342 (17.9%)	0.08	133 (18.0%)	301 (16.2%)	0.08
Quintile 4	0.01	124 (16.0%)	318 (16.6%)	0.02	124 (16.8%)	340 (18.3%)	0.02
Quintile 5 (highest)	0.02	123 (15.9%)	232 (12.1%)	0.04	96 (13.0%)	199 (10.7%)	0.04
**Region of Origin^**							
Caribbean	-	40 (5.2%)	160 (8.4%)	0.04	<6	5-10	0.05
Central America	-	<6 (0.5%)	15-20 (0.8%)	0.03	<6	15-20	0.03
East Africa	-	24 (3.1%)	49 (2.6%)	0.03	<6	5-10	0.07
East Asia	-	126 (16.3%)	334 (17.5%)	0.03	316 (42.7%)	742 (40.0%)	0.05
Eastern Europe	-	50 (6.5%)	114 (6.0%)	0.02	96 (13.0%)	266 (14.4%)	0.04
Middle East	-	67 (8.7%)	123 (6.4%)	0.08	41 (5.5%)	113 (6.1%)	0.02
North Africa	-	11 (1.4%)	21 91.1%)	0.03	<6	0	0.05
North America	-	33 (4.3%)	69 (3.6%)	0.03	0 (0.0%)	0 (0.0%)	-
South America	-	47 (6.1%)	89 (4.7%)	0.06	10 (1.4%)	30 (1.6%)	0.02
South Asia	-	103 (13.3%)	235 (12.3%)	0.03	62 (8.4%)	199 (10.7%)	0.08
Southeast Asia	-	131 (16.9%)	369 (19.3%)	0.06	80 (10.8%)	187 (10.1%)	0.02
Southern Africa	-	<6	<6	0.02	0 (0.0%)	0 (0.0%)	-
Southern Europe	-	17 (2.2%)	45 (2.4%)	0.01	28 (3.8%)	67 (3.6%)	0.01
USSR (former)	-	37 (4.8%)	93 (4.9%)	0.00	48 (6.5%)	92 (5.0%)	0.07
United Kingdom	-	<6 (0.1%0	0 (0.0%)	0.05	0 (0.0%)	0 (0.0%)	-
Western Africa	-	0 (0.0%0	6 (0.3%)	0.08	0 (0.0%)	0 (0.0%)	-
Western Europe	-	59 (7.6%)	114 (6.0%)	0.07	<6	5-10	0.04
Yugoslavia (former)	-	21 (2.7%)	72 (3.8%)	0.06	50 (6.8%)	117 (6.3%)	0.02
**No. PCP visits 6–30 months < index—patient’s usual provider of care^**							
Level 1 (0 - 10 visits)	0.26	334 (43.2%)	1,209 (63.2%)	0.19	284 (38.4%)	1,205 (65.0%)	0.19
Level 2 (11 - 18 visits)	0.08	248 (32.0%)	537 (28.1%)	0.02	251 (33.9%)	496 (26.8%)	0.02
Level 3 (19 - 30 visits)	0.18	111 (14.3%)	107 (5.6%)	0.20	113 (15.3%)	101 (5.5%)	0.20
Level 4 (31 - 50 visits)	0.22	81 (10.5%)	60 (3.1%)	0.20	92 (12.4%)	52 (2.8%)	0.20

**Institutional data requirements do not allow the reporting of any cell size less than 6, or reporting of any cell that would allow the calculation of another cell with size of less than 6

^Variables included in the full adjusted model

In both the unadjusted and fully adjusted models (Table 4), the stage of diagnosis was not associated with official language proficiency (ARR: 0.98; CI: 0.94–1.01 in the fully adjusted model). A lower income level was associated with the stage of diagnosis, where patients with income quintile level 1 (ARR: 1.08; 95% CI: 1.02–1.15), level 2 (ARR: 1.10; 95% CI: 1.04–1.17), and level 4 (ARR: 1.08; 95% CI: 1.01–1.15) were more likely to be diagnosed at a late stage, compared to income quintile level 5 (the highest income level). Region of origin was not strongly associated with stage of diagnosis, except for patients from the Caribbean (ARR: 1.16; 95% CI: 1.05–1.29) and South Asia (ARR: 1.10; 95% CI: 1.02–1.19), who were more likely to be diagnosed at a late stage. Lastly, patients with more primary care visits were more likely to be diagnosed at an early stage. No notable differences were observed after stratification by age or sex in the multivariable Poisson regression model.

**Table 4:** Multivariable model using Poisson regression. Adjusted relative risks represent late vs. early stage of diagnosis

Variables	Relative Risk (95% Confidence Interval)
**Unadjusted**	
Immigrant Language	1.00 (0.96-1.03)
**Full Model**	
Immigrant Language^	0.98 (0.94-1.01)
Male (vs. Female)^	1.11 (1.07-1.15)
Age (45-54)^	1.02 (0.94-1.11)
Age (55-64)^	1.00 (0.93-1.08)
Age (65-74)^	0.93 (0.86-1.00)
Age (75-84)^	0.95 (0.88-1.02)
**Neighborhood Income Quintile (Quintile 5 as the reference group)^**	
Quintile 1 (lowest)	1.08 (1.02-1.15)
Quintile 2	1.10 (1.04-1.17)
Quintile 3	1.06 (0.99-1.13)
Quintile 4	1.08 (1.01-1.15)
**No. PCP Visits in 2 years prior to diagnosis (level 1 as the reference group)^**	
Level 2 (11-18)	0.85 (0.82-0.89)
Level 3 (19-30)	0.62 (0.57-0.67)
Level 4 (31-50)	0.51 (0.46-0.56)
**Region of Origin (Yugoslavia (former) as the reference group)^**	
Caribbean	1.16 (1.05-1.29)
Central America	1.08 (0.88-1.32)
East Africa	1.03 (0.88-1.19)
East Asia	1.04 (0.97-1.11)
Eastern Europe	1.02 (0.94-1.10)
Middle East	1.05 (0.96-1.14)
North Africa	0.99 (0.78-1.25)
North America	1.00 (0.87-1.15)
South America	1.00 (0.90-1.12)
South Asia	1.10 (1.02–1.19)
Southeast Asia	1.04 (0.97-1.12)
Southern Africa	0.94 (0.58-1.51)
Southern Europe	0.99 (0.89-1.11)
United Kingdom	*
Western Africa	1.45 (0.94-2.24)
Western Europe	1.00 (0.89-1.11)
USSR (former)	*

*Not applicable due to sparse data

^Variables included in the full adjusted model

In MLE analyses (Table 5), we found that newly arrived English/French speaking immigrants were significantly more likely than more distantly arrived immigrants were to be diagnosed at a late stage (OR: 0.73; 95% CI: 0.56–0.95). No notable differences were observed among non-English/French speaking immigrants.

**Table 5:** Analysis of maximum likelihood estimates for time since arrival. Odds ratio represent late vs. early stage of diagnosis

Variables	Odds ratio (95% Confidence Interval)
**English/French speaking (Level 4 as reference group)**	
Level 0 (most recent time since arrival)	0.73 (0.56–0.95)
Level 1	0.87 (0.67–1.14)
Level 2	1.16 (0.88–1.52)
Level 3	1.06 (0.81–1.39
**Non English/French speaking (Level 4 as reference group)**	
Level 0	1.12 (0.85–1.48
Level 1	1.03 (0.79–1.33)
Level 2	1.07 (0.82–1.39)
Level 3	1.10 (0.84–1.43)

A descriptive table including long-term residents stratified by early vs. late-stage diagnosis and distribution of years since landing is included in the appendix.

## Discussion

In this retrospective cohort study of 96,613 incident lung cancer cases between 2010 and 2020 in Ontario, Canada, we found that 57.7% of individuals were diagnosed at a late stage. Among the 6,554 immigrants, 57.5% were diagnosed at a late stage. There was no significant difference in the lung cancer stage at diagnosis between non-English/French speaking immigrants and English/French speaking immigrants, nor was the diagnostic stage associated with the region of origin, with the exceptions of the Caribbean and South Asia. However, newly arrived English/French speaking immigrants were significantly more likely than more distantly arrived immigrants to be diagnosed at a late stage.

Our findings that 57.7% of patients are diagnosed at a late stage and is more pronounced for recent immigrants, reiterates the need for culturally sensitive targeted outreach for lung cancer awareness in Ontario. Lung cancer is typically diagnosed symptomatically; however, the lack of specific symptoms and overlap with other conditions can contribute to a delay in diagnosis [25]. Currently, there are only two organized lung cancer screening programs in Canada: British Columbia and Ontario. Pilot studies are currently underway in Alberta and Quebec to investigate the feasibility of implementing organized screening programs. Like those in British Columbia and Ontario, they are limited to eligible high-risk people [26]. To be eligible for lung cancer screening in British Columbia and Ontario, participants must be between the ages of 55 and 74 years, be a current or former smoker of 20 years or more and not currently experiencing symptoms [27, 28]. Given the rising proportion of lung cancer cases among non-smokers [29], lung screening programs may benefit from eligibility criteria.

In fully adjusted models, we found that individuals residing in a neighborhood with a lower average income quintile were more likely to be diagnosed at a late stage than those who reside in a neighborhood with a higher income quintile after accounting for potential language barriers, suggesting economic barriers in accessing screening and treatment despite a universal healthcare system in Ontario and Canada. English/French speaking immigrants were slightly more likely than non-English/French speaking immigrants to live in a neighborhood with a higher income quintile. Nevertheless, both immigrant groups are disproportionately more likely to live in the lowest income quintile than long-term residents are, putting them at an increased risk for late-stage diagnosis. We also found that more primary care contact was associated with earlier stages of cancer diagnosis. However, both immigrant groups had similar distributions, suggesting that a lack of official language fluency is not a barrier to primary care access.

We found that immigrants from the Caribbean and South Asia are more likely to be diagnosed with lung cancer at a late stage. Immigration from the Caribbean and South Asia has been steadily increasing since the 1970 s [30]. Although most of them belonged to the English/French speaking group in our study (96% and 56% respectively), both groups were found to be significantly more likely to be diagnosed at a late stage. A previous paper by our group in 2022 on a similar cohort within the same province found that more than 50% of immigrants from the Caribbean and South Asia belong to the lowest 2 income quintiles (57.0% and 51.9%, respectively) [31]. This disproportionate distribution may in part account for our finding that residents of neighborhoods with a lower average income quintile are more likely to be diagnosed at a late stage. Similarly, a 2022 study by Fry et al. revealed that Caribbean and South Asian patients in England were more likely to be diagnosed with late-stage breast, uterine, or colon cancer than their white British counterparts were [32]. These findings suggest that Caribbean and South Asian immigrants may be affected by other factors and highlight the need for further research and targeted lung cancer screening campaigns.

Adenocarcinoma was the most prevalent lung cancer type, particularly among English/French speaking immigrants, and is known to be the most common subtype to be diagnosed in non-smokers. Tobacco smoking is still the main risk factor for lung cancer [26] and was more common among men than women at 12.9% and 9.1%, respectively, in 2022 [33]. Canadian immigrants and those in a higher income quintile are generally less likely to smoke than non-immigrants and those in a lower income quintile are [34, 35]. As such, it is unsurprising that we found an association between the lower neighborhood income quintile and sex with late stage diagnosis. Unfortunately, individual-level smoking status was not available from ICES. Smoking cessation is a key target of Ontario’s current lung cancer screening program, and the relationship between smoking cessation and lung cancer stage at diagnosis among different immigrant groups could be an area for future research.

Previous studies have shown that older age at immigration is generally correlated with reduced English proficiency in adulthood, primarily due to not participating in public schooling [36]. Older adults who speak English fluently may also have reduced proficiency over time: aphasia, or the loss of language, is common in patients with dementia [37, 38]. As such, it is unsurprising that MLE analyses showed that time since arrival had no significant effect on cancer staging in non-English/French fluent immigrants; not only are they less likely to have learned English to a high proficiency since arrival, but they are also more likely to have reduced proficiency over time. In contrast, the significant difference observed in English/French speaking immigrants may reflect greater familiarity with the healthcare system over time. Increasing healthcare literacy among new immigrants may be beneficial for improving the prognosis and overall health of patients with lung cancer.

This study has several limitations. First, immigrants who arrived before 1985 or landed in another province are not captured in the IRCC-PR database and are thus classified as long-term residents. Similarly, we were unable to capture the exact age at immigration due to ICES cell suppression policies. As participation in public schooling is generally correlated with English proficiency in adulthood [36], the unknown age at immigration may have negatively affected language categorization. In addition, official language proficiency in a casual or workplace setting may not translate to language proficiency in a medical setting, and as such, the English/French speaking group may be overrepresented. Second, our use of broader regions of origin obscured unique aspects about their country of origin. For example, a 2021 study by Gallus et al.. reported smoking rates ranging from 18.9% in Italy to 36.8% in Portugal. In our study, both countries would fall into the same region of Southern Europe [39]. Additionally, the small number of patients in each region of origin resulted in limited group sizes, causing class imbalance in the multivariable regression analysis. Third, the cancer type was unknown for more than 17.8% of the study population; thus, our findings regarding cancer type must be interpreted with caution. Fourth, we were unable to capture any information about cigarette use, vaping, e-cigarette use, or other risk factors for lung cancer, such as exposure to asbestos, second-hand smoke, or family history. Fifth, we had missing staging data for 17.8% of cases. As there were similar rates of missing data in all groups, we assumed that data was missing at random. However, further analyses could be conducted in future work to explore alternative assumptions and the impact of missing data on these findings. Finally, since rural residents have differing access to healthcare services [40] and more than 90% of Canadian immigrants live in urban settings [22], we limited the study to urban residents. As such, our results cannot be generalized to non-urban settings. Despite these limitations, our study is strengthened by its use of census level high-quality demographic and healthcare data.

As lung cancer screening programs roll out across Canada, future research should continue to monitor for inequalities in the stages of diagnosis and screening rates to target recruiting and marketing efforts. Research has shown disproportionately low cancer screening rates among racialized communities, immigrants, and other underserved communities [5, 8, 9, 41–45]. Data also suggest that there are considerable decreases in the number of cancer diagnoses due to the COVID-19 pandemic, and the long-term impact of COVID-19 on lung cancer outcomes in Canadians may not be known for years [26]. It is crucial that clear and efficient diagnostic pathways are developed so that people who do not qualify or are unable to access lung cancer screening but present with worrisome symptoms associated with lung cancer are able to access the care they need.

## Conclusions

Lung cancer in Ontario is frequently diagnosed at a late stage. English/French language proficiency is not associated with a late-stage diagnosis among Ontario immigrants; however, men, those in a lower neighborhood income quintile, and Caribbean and South Asian immigrants have a greater likelihood of being diagnosed at a later stage. As lung cancer screening programs are beginning to be implemented across Canada, culturally and linguistically sensitive targeted outreach to immigrants and underserved communities needs to be prioritized.

## Supplementary Information

Supplementary Material 1

## Data Availability

The dataset from this study is held securely in coded form at the ICES. While data sharing agreements prohibit the ICES from making the dataset publicly available, access may be granted to those who meet prespecified criteria for confidential access, available at www.ices.on.ca/DAS. The full dataset creation plan and underlying analytic code are available from the authors upon request, understanding that the computer programs may rely upon coding templates or macros that are unique to the ICES and are therefore either inaccessible or may require modification.

## Abbreviations

ARR: Adjusted relative risk
CI: Confidence interval
OR: Odds ratio
MLE: Maximum Likelihood Estimates
