# Supplementary material for: Lung cancer stage at diagnosis and immigrant English/French language proficiency: a retrospective population level cohort study of urban residents in Ontario, Canada
Source: BMC Cancer. 2025 Sep 30;25:1452. doi: 10.1186/s12885-025-14666-z (PMC12481846; doi:10.1186/s12885-025-14666-z)
Supplement: Supplementary file 1 — Supplementary Material 1 [file 12885_2025_14666_MOESM1_ESM.docx]

**Appendix 1**

**Table 1: Descriptive characteristics of study cohort stratified by stage of diagnosis (early vs. late) and immigrant status (excluding patients with missing lung cancer stage)**

***Institutional data requirements do not allow the reporting of any cell size less than 6, or reporting of any cell that would allow the calculation of another cell with size of less than 6.*

| **Characteristics** | **Long-term Residents**  **(n=74,137)** | | **S.D.**  **(Late – Early stage for long-term residents)** | | **English/French Speaking Immigrants**  **(n=2,687)** | | **S.D.**  **(Late – Early stage for English/French Speaking immigrants** | **Non-English/French Speaking Immigrants**  **(n=2,594)** | | **S.D.**  **(Late – Early stage for Non-English/French Speaking immigrants)** |
| --- | --- | --- | --- | --- | --- | --- | --- | --- | --- | --- |
|  | **Early**  **Stage**  **(n=22,203)** | **Late**  **Stage**  **(n=51,934)** |  |  | **Early**  **Stage**  **(n=774)** | **Late**  **Stage**  **(n=1,913)** |  | **Early**  **Stage**  **(n=740)** | **Late**  **Stage**  **(n=1,854)** |  |
| **Sex** | | | | | | | | | | |
| Female | 12,103  (54.5%) | 24,917  (48.0%) | 0.09 | | 362  (46.8%) | 677  (35.4%) | 0.18 | 363  (49.1%) | 728  (39.3%) | 0.18 |
| Male | 10,100  (45.5%) | 27,017  (52.0%) | | 0.09 | 412  (53.2%) | 1,236  (64.6%) | 0.18 | 377  (50.9%) | 1,126  (60.7%) | 0.18 |
| **Age Group** | | | | | | | | | | |
| 45-54 | 980  (4.4%) | 3,308  (6.4%) | 0.09 | | 120  (15.5%) | 355  (18.6%) | 0.13 | 72  (9.7%) | 187  (10.1%) | 0.13 |
| 55-64 | 4,271  (19.2%) | 11,733  (22.6%) | 0.12 | | 230  (29.7%) | 625  (32.7%) | 0.07 | 153  (20.7%) | 426  (23.0%) | 0.07 |
| 65-74 | 8,059  (36.3%) | 17,651  (34.0%) | 0.01 | | 239  (30.9%) | 507  (26.5%) | 0.08 | 230  (31.1%) | 514  (27.7%) | 0.08 |
| 75-84 | 7,084  (31.9%) | 14,631  (28.2%) | 0.08 | | 155  (20.0%) | 332 (17.4%) | 0.06 | 228  (30.8%) | 569  (30.7%) | 0.06 |
| 85+ | 1,809  (8.2%) | 4,611  (8.9%) | 0.11 | | 30  (3.9%) | 94  (4.9%) | 0.07 | 57  (7.7%) | 158  (8.5%) | 0.07 |
| **Neighborhood Income Quintile** | | | | | | | | | | |
| Quintile 1 (lowest) | 5,156  (23.2%) | 12,680  (24.4%) | 0.02 | | 228  (29.5%) | 555  (29.0%) | 0.01 | 222  (30.0%) | 564  (30.4%) | 0.01 |
| Quintile 2 | 4,896  (22.1%) | 11,884  (22.9%) | 0.02 | | 162  (20.9%) | 466  (24.4%) | 0.08 | 165  (22.3%) | 450  (24.2%) | 0.08 |
| Quintile 3 | 4,406  (19.8%) | 10,049  (19.4%) | 0.01 | | 137  (17.7%) | 342  (17.9%) | 0.08 | 133  (18.0%) | 301  (16.2%) | 0.08 |
| Quintile 4 | 3,959  (17.8%) | 9,060  (17.5%) | 0.01 | | 124  (16.0%) | 318  (16.6%) | 0.02 | 124  (16.8%) | 340  (18.3%) | 0.02 |
| Quintile 5 (highest) | 3,786  (17.1%) | 8,261  (15.9%) | 0.02 | | 123  (15.9%) | 232  (12.1%) | 0.04 | 96  (13.0%) | 199  (10.7%) | 0.04 |
| **Lung Cancer Type** | | | | | | | | | | |
| Adenocarcinoma | 6,283  (28.3%) | 18,309  (35.3%) | 0.22 | | 240  (31.1%) | 973  (50.9%) | 0.41 | 242  (32.7%) | 852  (46.0%) | 0.35 |
| Others | 10,833  (48.8%) | 18,275  (35.2%) | 0.42 | | 438  (56.6%) | 554  (29.0%) | 0.6 | 374  (50.5%) | 543  (29.3%) | 0.57 |
| Small Cell | 757  (3.41%) | 7,671  (14.8%) | 0.36 | | 18  (2.3%) | 188  (9.8%) | 0.28 | 16  (2.2%) | 200  (10.8%) | 0.29 |
| Squamous | 4,330  (19.5%) | 7,679  (14.8%) | 0.01 | | 78  (10.1%) | 198  (10.4%) | 0.06 | 108  (14.6%) | 259  (14.0%) | 0.10 |
| **Region of Origin** | | | | | | | | | | |
| Caribbean | - | - | - | | 40  (5.2%) | 160  (8.4%) | 0.04 | <6 | 5-10 | 0.05 |
| Central America | - | - | - | | <6  (0.5%) | 15-20  (0.8%) | 0.03 | <6 | 15-20 | 0.03 |
| East Africa | - | - | - | | 24  (3.1%) | 49  (2.6%) | 0.03 | <6 | 5-10 | 0.07 |
| East Asia | - | - | - | | 126  (16.3%) | 334  (17.5%) | 0.03 | 316  (42.7%) | 742  (40.0%) | 0.05 |
| Eastern Europe | - | - | - | | 50  (6.5%) | 114  (6.0%) | 0.02 | 96  (13.0%) | 266  (14.4%) | 0.04 |
| Middle East | - | - | - | | 67  (8.7%) | 123  (6.4%) | 0.08 | 41  (5.5%) | 113  (6.1%) | 0.02 |
| North Africa | - | - | - | | 11  (1.4%) | 21  91.1%) | 0.03 | <6 | 0 | 0.05 |
| North America | - | - | - | | 33  (4.3%) | 69  (3.6%) | 0.03 | 0  (0.0%) | 0  (0.0%) | - |
| South America | - | - | - | | 47  (6.1%) | 89  (4.7%) | 0.06 | 10  (1.4%) | 30  (1.6%) | 0.02 |
| South Asia | - | - | - | | 103  (13.3%) | 235  (12.3%) | 0.03 | 62  (8.4%) | 199  (10.7%) | 0.08 |
| Southeast Asia | - | - | - | | 131  (16.9%) | 369  (19.3%) | 0.06 | 80  (10.8%) | 187  (10.1%) | 0.02 |
| Southern Africa | - | - | - | | <6 | <6 | 0.02 | 0  (0.0%) | 0  (0.0%) | - |
| Southern Europe | - | - | - | | 17  (2.2%) | 45  (2.4%) | 0.01 | 28  (3.8%) | 67  (3.6%) | 0.01 |
| USSR (former) | - | - | - | | 37  (4.8%) | 93  (4.9%) | 0.00 | 48  (6.5%) | 92  (5.0%) | 0.07 |
| United Kingdom | - | - | - | | <6  (0.1%0 | 0  (0.0%) | 0.05 | 0  (0.0%) | 0  (0.0%) | - |
| Western Africa | - | - | - | | 0  (0.0%0 | 6  (0.3%) | 0.08 | 0  (0.0%) | 0  (0.0%) | - |
| Western Europe | - | - | - | | 59  (7.6%) | 114  (6.0%) | 0.07 | <6 | 5-10 | 0.04 |
| Yugoslavia (former) | - | - | - | | 21  (2.7%) | 72  (3.8%) | 0.06 | 50  (6.8%) | 117  (6.3%) | 0.02 |
| **No. PCP visits 0–24 months < index—patient’s usual provider of care** | | | | | | | | | | |
| Level 1 (lowest number of visits) | 10,695  (48.2%) | 37,020  (71.3%) | 0.26 | | 334  (43.2%) | 1,209  (63.2%) | 0.19 | 284  (38.4%) | 1,205  (65.0%) | 0.19 |
| Level 2 | 6,959  (31.3%) | 11,685  (22.5%) | 0.08 | | 248  (32.0%) | 537  (28.1%) | 0.02 | 251  (33.9%) | 496  (26.8%) | 0.02 |
| Level 3 | 2,650  (11.9%) | 2,240  (4.3%) | 0.18 | | 111  (14.3%) | 107  (5.6%) | 0.20 | 113  (15.3%) | 101  (5.5%) | 0.20 |
| Level 4  (highest number of visits) | 1,899  (8.6%) | 989  (1.9%) | 0.22 | | 81  (10.5%) | 60  (3.1%) | 0.20 | 92  (12.4%) | 52  (2.8%) | 0.20 |
